# A Case of a Missing Proximal Humerus

**DOI:** 10.5811/cpcem.2020.6.48396

**Published:** 2020-07-30

**Authors:** Jessica Lynn Williams, Steven M. Hochman

**Affiliations:** Saint Joseph’s University Medical Center, Emergency Department, Paterson, New Jersey

**Keywords:** proximal humerus, metastatic bone lesions, renal cell carcinoma

## Abstract

**Case Presentation:**

In this case, we demonstrate how a small radiolucency in the proximal humerus can progress to an even larger problem within a few months in a patient without follow-up. Our patient’s ultimate diagnosis was renal cell carcinoma with metastasis to the right proximal humerus, completely obliterating the affected bone.

**Discussion:**

In many underserved communities, patients have limited access to medical care, particularly specialty care. These patients often present to the emergency department and are unable to acquire appropriate follow-up. This situation illustrates the social issues that our patients face every day affecting their access to healthcare and ultimately necessary medical treatment.

## CASE PRESENTATION

**Image 1:**
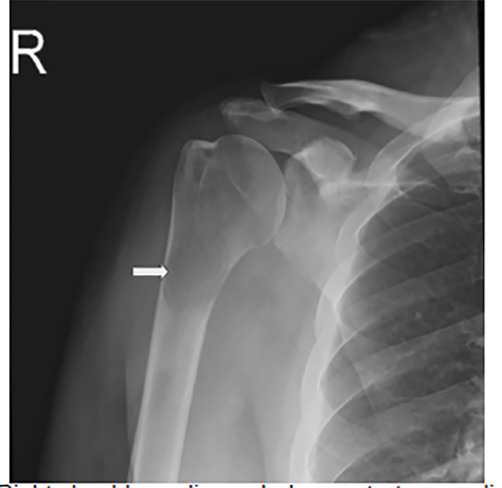
Right shoulder radiograph demonstrates a radiolucency (arrow) in the right proximal humerus on the initial visit.

A 56-year-old male with a history of alcoholic liver cirrhosis presented to the emergency department (ED) for worsening atraumatic right proximal arm pain. His examination was remarkable for limited active and passive range of motion of the right shoulder. He had presented to an affiliated ED three months prior for similar complaints. At that time, radiographs demonstrated a radiolucency in the right proximal humerus (Image 1). Computed tomography on the same date demonstrated a metastatic or a primary bone lesion.

**Image 2:**
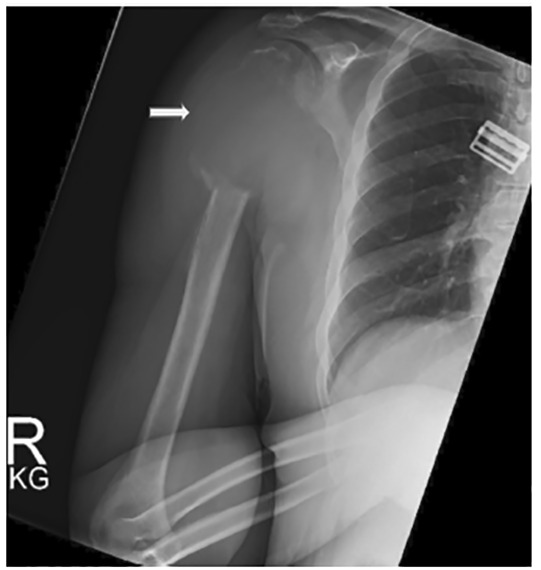
Right humerus radiograph demonstrates an area of presumed soft-tissue mass causing bony destruction of the right proximal humerus (arrow) three months later.

**Image 3:**
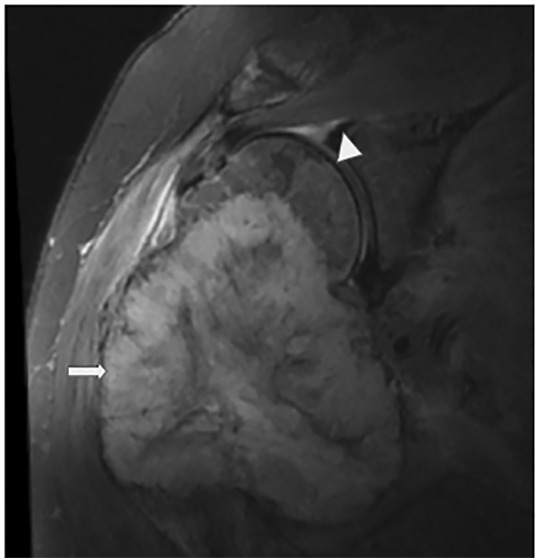
Magnetic resonance imaging of the right humerus demonstrates a large, heterogeneous mass (arrow) adjacent to the right humeral neck and head, which extends peripherally into the soft tissues, representing malignancy. Arrowhead denotes the normal humeral head.

The patient was discharged and instructed to follow up as an outpatient, but was unable to do so. The radiograph of the right humerus on the current visit demonstrated a large, soft tissue lytic mass (Image 2). The patient was admitted to the hospital and diagnosed with renal cell carcinoma (RCC) of the right kidney with metastasis to the humerus. Magnetic resonance imaging of the right humerus four days after admission can be seen in Image 3. The patient underwent right radical resection of the right proximal humerus mass, reverse total shoulder arthroplasty and rotator cuff repair at another institution. Pathology reports confirmed the humeral mass was metastatic RCC. The patient was started on infusion therapy.

## DISCUSSION

RCC is responsible for 3% of all cancers.^1^ Bone metastasis is most commonly from breast, prostate, and lungs.^2^ However, 25–30% of RCC tumors metastasize to bone.^1^ Emergency physicians should have a low threshold for obtaining radiographs in patients with atraumatic pain to rule out pathologic lesions. With a five-year RCC survival rate of less than 50%, early detection and initiation of treatment are essential, as earlier stages of cancer have better survival rates and treatment options. If patients lack follow-up or insurance, emergency providers must be diligent to provide patient education and assist in arranging follow-up for a better chance of survival.

**CPC-EM Capsule**

What do we already know about this clinical entity?

Once cancer becomes stages 3 or 4 with metastasis, there are fewer treatment options and lower survival rates.

What is the major impact of the image(s)?

Subtle nonspecific findings on plain films can be early indications of pathology that can progress rapidly, reinforcing the importance of early diagnosis and treatment.

How might this improve emergency medicine practice?

Imaging should be obtained for atraumatic pain in patients with limited access to follow-up care who present with insidious onset or whose pain occurs at night or is not relieved by conservative treatment.
